# An innovative diagnostic technology for the codon mutation C580Y in *kelch13* of *Plasmodium falciparum* with MinION nanopore sequencer

**DOI:** 10.1186/s12936-018-2362-x

**Published:** 2018-05-29

**Authors:** Kazuo Imai, Norihito Tarumoto, Lucky Ronald Runtuwene, Jun Sakai, Kyoko Hayashida, Yuki Eshita, Ryuichiro Maeda, Josef Tuda, Hideaki Ohno, Takashi Murakami, Shigefumi Maesaki, Yutaka Suzuki, Junya Yamagishi, Takuya Maeda

**Affiliations:** 1 Department of Infectious Disease and Infection Control, Saitama Medical University, 38 Morohongo, Moroyama-machi, Iruma-gun, Saitama 350-0495 Japan; 2 Center for Clinical Infectious Diseases and Research, Saitama Medical University, 38 Morohongo, Moroyama-machi, Iruma-gun, Saitama 350-0495 Japan; 3 Department of Computational Biology and Medical Science, Graduate School of Frontier Sciences, The University of Tokyo, 5-1-5 Kashiwanoha, Kashiwa, Chiba 277-8562 Japan; 4 Research Center for Zoonosis Control, Hokkaido University, North 20, West 10 Kita-ku, Sapporo, Hokkaido 001-0020 Japan; 5 Faculty of Medicine, Oita University, 1-1 Hasama-machi, Yufu, Oita 879-5593 Japan; 6 Department of Medical Entomology, Faculty of Tropical Medicine, Mahidol University, 420/6 Ratchawithi Road, Thung Phaya, Ratchathewi, Bangkok, 10400 Thailand; 7 Research Institute for Microbial Diseases, Osaka University, 3-1 Yamadaoka, Suita, Osaka 565-0871 Japan; 8 Division of Biomedical Sciences, Department of Basic Veterinary Medicine, Obihiro University of Agriculture and Veterinary Medicine, 2-11 Inada-cho, Obihiro, Hokkaido 080-8555 Japan; 9 Department of Parasitology, Faculty of Medicine, Sam Ratulangi University, Kampus Unsrat, Bahu Manado, 95115 Indonesia; 10 Department of Infectious Diseases and Infection Control, Saitama Medical Center, Saitama Medical University, 1981 Kamoda, Kawagoe, Saitama 350-8550 Japan; 11 Department of Microbiology, Saitama Medical University, 38 Morohongo, Moroyama-machi, Iruma-gun, Saitama 350-0495 Japan; 12 Global Station for Zoonosis Control, GI-CoRE, Hokkaido University, North 20, West 10 Kita-ku, Sapporo, Hokkaido 001-0020 Japan

**Keywords:** Malaria, *Plasmodium falciparum*, *Artemisinin resistance*, LAMP, Nanopore sequencer, MinION™, *kelch 13*

## Abstract

**Background:**

The recent spread of artemisinin (ART)-resistant *Plasmodium falciparum* represents an emerging global threat to public health. In Southeast Asia, the C580Y mutation of *kelch13* (*k13*) is the dominant mutation of ART-resistant *P. falciparum.* Therefore, a simple method for the detection of C580Y mutation is urgently needed to enable widespread routine surveillance in the field. The aim of this study is to develop a new diagnostic procedure for the C580Y mutation using loop-mediated isothermal amplification (LAMP) combined with the MinION nanopore sequencer.

**Results:**

A LAMP assay for the *k13* gene of *P. falciparum* to detect the C580Y mutation was successfully developed. The detection limit of this procedure was 10 copies of the reference plasmid harboring the *k13* gene within 60 min. Thereafter, amplicon sequencing of the LAMP products using the MinION nanopore sequencer was performed to clarify the nucleotide sequences of the gene. The C580Y mutation was identified based on the sequence data collected from MinION reads 30 min after the start of sequencing. Further, clinical evaluation of the LAMP assay in 34 human blood samples collected from patients with *P. falciparum* malaria in Indonesia revealed a positive detection rate of 100%. All LAMP amplicons of up to 12 specimens were simultaneously sequenced using MinION. The results of sequencing were consistent with those of the conventional PCR and Sanger sequencing protocol. All procedures from DNA extraction to variant calling were completed within 3 h. The C580Y mutation was not found among these 34 *P. falciparum* isolates in Indonesia.

**Conclusions:**

An innovative method combining LAMP and MinION will enable simple, rapid, and high-sensitivity detection of the C580Y mutation of *P. falciparum*, even in resource-limited situations in developing countries.

**Electronic supplementary material:**

The online version of this article (10.1186/s12936-018-2362-x) contains supplementary material, which is available to authorized users.

## Background

Malaria is a mosquito-borne infectious disease and one of the most severe and prevalent public health problems in tropical and subtropical areas worldwide. In recent years, the spread of artemisinin (ART)-resistant *Plasmodium falciparum* malaria, which is defined as delayed parasite clearance from the blood following appropriate treatment with ART monotherapy or ART-based combination therapy (ACT) [1], has gained global attention, particularly in Southeast Asia and southern China [2–4]. The World Health Organization (WHO) currently recommends ACT instead of artesunate monotherapy as the first-line treatment for suspected *P. falciparum* malaria infections, and has urgently proposed a global plan for the monitoring and surveillance of ART-resistant *P. falciparum* to assess the threat of emerging resistant strains [5].

A molecular marker of ART resistance in *P. falciparum* has not been completely identified or validated; however, mutations in the *kelch13* (*k13*)-propeller gene are thought to be associated with ART resistance based on in vitro and in vivo investigations [1]. Although amino acids substitutions N458Y, Y493H, R539T, I543T, R561H, and C580Y could all be associated with ART-resistance and reduce the cure rate following ACT treatment, C580Y is thought to be the dominant mutation and a potential drug resistance marker, especially in Southeast Asia [6–9]. Recently, ART-resistant strains of *P. falciparum* with C580Y mutation have spread across Cambodia, northeastern Thailand, and southern Laos. Simultaneously, the mutation could have developed resistance to piperaquine—one of the ACT partner drugs—because this drug is related to high rates of failure in the treatment of malaria [8]. Thus, there is an urgent need to strengthen the surveillance and elimination of ART-resistant *P. falciparum* C580Y mutation in Southeast Asia.

Point mutations and their prevalence can be confirmed by polymerase chain reaction (PCR) amplification and Sanger sequencing, which are widely used and well established. However, these techniques are unsuitable in malaria-endemic areas due to their high cost and need for sophisticated laboratories, and they cannot keep up with the current demand for field-based assays. Clinical samples need to be stored by appropriate protocols and transferred to laboratories from remote locations, which creates several disadvantages, including the cost of transportation, transfer time, and potential loss of clinical samples. Therefore, a simple and easy-to-perform molecular genotyping method is urgently required to accurately assess the distribution of ART-resistant *P. falciparum* in malaria-endemic areas.

Loop-mediated isothermal amplification (LAMP) is an isothermal nucleic acid amplification technique that has some operational advantages over conventional PCR procedures because it is fast, easy to use, and cost-effective [10]. Amplification is mediated by a loop structure and relies on an auto-cycling procedure performed with strand-displacement of *Bst* DNA polymerase under isothermal conditions. Furthermore, the amplification products in LAMP reactions can be directly judged by turbidity or changes in fluorescence with the naked eye. Given these properties, the LAMP method is expected to become an important and widely-used clinical diagnostic technique in point-of-care testing for infectious diseases and will require only limited equipment and labor. The advantages of LAMP are particularly relevant in areas with limited resources [11, 12]. Recently, Loopamp™ MALARIA Pan/Pf Detection Kit (Eiken Chemical Co., Ltd., Tokyo, Japan) has become commercially available for the detection of *Plasmodium* parasites.

The MinION nanopore sequencer is a pocket-sized and USB-connected portable real-time sequencer developed by Oxford Nanopore Technologies (ONT; Oxford, UK). The advantages of MinION include the simple and rapid preparation of samples, portability, real-time sequencing, relatively low cost, and the need for minimal equipment and personnel. Recently, MinION has been shown to be an alternative method for whole genome sequencing that can be used for genomic surveillance and infection control in hospitals by enabling the rapid identification of pathogens, including Ebola virus, in areas with limited resources [13, 14]. In addition, an innovative diagnostic technology using the LAMP assay combined with MinION, which is rapid, simple, highly sensitive, and cost-effective, has been introduced for the serotyping of dengue virus and genotyping of *Plasmodium* parasites [15, 16].

In this study, a single nucleotide polymorphism genotyping method for C580Y in *k13* of *P. falciparum* with clinical specimens using the LAMP assay combined with MinION nanopore sequencing was developed. This is a quick, sensitive, and simple method that requires minimal equipment and personnel.

## Methods

### Design of LAMP primers

**Fig. 1:**
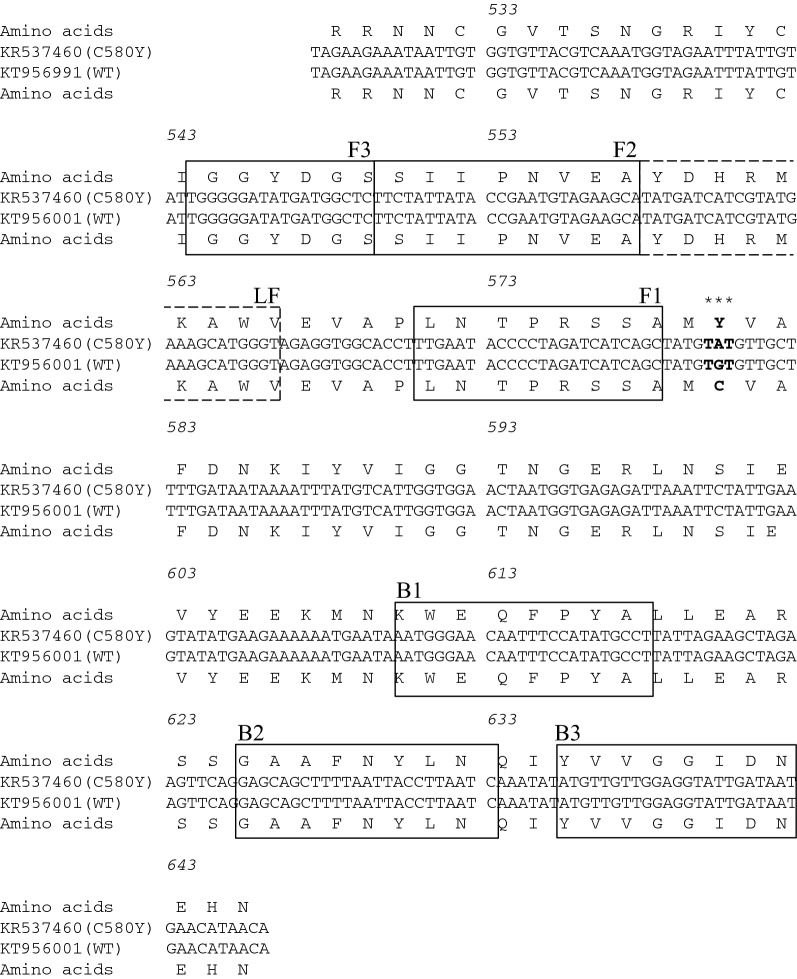
Alignment of the partial sequences of the *kelch13* of *Plasmodium falciparum*, which was constructed within the pEX-A2J1 plasmid. The constructed sets of LAMP primers are shown as lines and boxes. Asterisks show the specific sequences located at the codon position of C580Y in artemisinin-resistant *P. falciparum*

The oligonucleotide LAMP primers for the detection of the codon mutation of C580Y were designed based on the sequences of *k13* to encompass C580Y within the F1–B1 primer pairs via online LAMP primer design software (PrimerExplorer 5, http://primerexplorer.jp/index.html; Eiken Chemical). Based on in silico analysis of the *k13* sequence in six human *Plasmodium* parasites (*P. falciparum*, *Plasmodium vivax*, *Plasmodium ovale curtisi*, *Plasmodium ovale wallikeri*, *Plasmodium knowlesi*, and *Plasmodium malariae*), these primers were located on diverse sequences for species-specific LAMP amplification (Additional file 1: Fig. S1). The sequences of each selected primer are given in Table 1 and their positions are shown in Fig. 1.

**Table 1:** Nucleotide sequences of the LAMP primers constructed for *kelch13* of *Plasmodium falciparum*

Primer	Sequence (5′–3′)	Length
LAMP primers		
F3	TGGGGGATATGATGGCTC	18
B3	ATTATCAATACCTCCAACAACAT	23
FIP	AGCTGATGATCTAGGGGTATTCAA-TTCTATTATACCGAATGTAGAAGCA	49
BIP	AATGGGAACAATTTCCATATGCCT-GATTAAGGTAATTAAAAGCTGCTC	48
LF	CCCATGCTTTCATACGATGATCATA	25

F3 and B3, outer primers; FIP and BIP, inner primers; LF, loop primer

The FIP primer consisted of F2 and the complementary strand (F1c)

The BIP primer consisted of B2 and the complementary strand (B1c)

### Plasmid construction

The templates used for the analytical LAMP reactions were the pEX-A2J1 plasmids harbouring partial sequences within the *k13* of *P. falciparum*, which were constructed by Eurofines Genomics Co., Ltd. (Tokyo, Japan) based on the reference sequences (GenBank accession numbers: *P. falciparum*, KT956001.1 and KR537460.1). The sequences of the constructed plasmids are summarized in Fig. 1. The plasmids were serially diluted tenfold and adjusted from 1.0 × 10^1^ to 1.0 × 10^5^ copies/μL to determine the detection threshold and specificity of the LAMP reactions. The sequences of the constructed plasmids were confirmed by the Sanger method using F3 and B3 primers (Table 1).

### LAMP reactions

The reactions were performed using a Loopamp DNA Amplification Reagent Kit (Eiken Chemical). Detection of the LAMP amplicons was performed by real-time measurements of turbidity using a Loopamp real-time turbidimeter LA-200 (Eiken Chemical) and visual observations of the colour changes with the naked eye under natural light. The final reaction volume was 25 μL, comprising 40 pmol of the FIP primer and 40 pmol of the BIP primer, 20 pmol of each loop primer, 5 pmol of F3 and B3 primers, 1 μL of the Bst DNA polymerase, reaction buffer (20 mM Tris–HCl, 10 mM KCl, 8 mM MgSO_4_, 10 mM (NH_4_)_2_SO_4_, 0.1% Tween 20, 0.8 M betaine, and 1.4 mM each dNTP), and 1 μL of plasmid DNA or 8 μL of genomic DNA samples. Reactions were carried out at 62, 63, 64, and 65 °C in duplicate to find the optimum temperature for the LAMP amplification.

### The detection limit and specificity of the LAMP assay

Ten-fold serial dilutions of each plasmid DNA ranging from 1.0 × 10^1^ to 1.0 × 10^5^ copies/μL were produced to determine the detection limit of the LAMP primers, and stored at − 20 °C in duplicate until use. Additionally, to evaluate the specificity of the test, seven different protozoa (*Toxoplasma gondii*, *Cryptosporidium parvum*, *Giardia intestinalis*, *Entamoeba histolytica, Leishmania donovani, Trypanosoma brucei rhodesiense*, and *Trypanosoma cruzi*) isolated by the National BioResource Project (http://www.nbrp.jp/) were also examined.

### Clinical samples and ethics

Clinical samples were obtained from Sam Ratulangi University in Manado and Bitung, North Sulawesi, Indonesia from August to December 2010. All patients were diagnosed based on symptoms, blood smears stained with Giemsa stain, and nested PCR according to previously reported protocols [17–19]. Peripheral blood was collected by FTA Elute cards (GE Healthcare Life Sciences, Little Chalfont, UK) from each patient and stored at room temperature.

Both the design and protocol of this study conformed to the Helsinki Declaration and were approved by the Institutional Ethics Committee. All samples were collected after written informed consent has been obtained. The methods of collection and analysis of the human samples were approved and cleared by the Institutional Ethical Review Board of Sam Ratulangi University and the University of Tokyo.

### DNA extraction from clinical samples

Genomic DNA was extracted following the manufacturer’s instructions for FTA Elute cards. Briefly, each disk with a diameter of 3.0 mm was cut from the blood spot areas and washed three times with 200 μL of distilled water. DNA was eluted in 30 μL of TE buffer (10 mM Tris–HCl, 0.1 mM EDTA, pH 8.0) with a heat block at 95 °C for 30 min. Finally, 8.0 μL of supernatant was used as template DNA for both LAMP assays.

### Sequencing of LAMP products by MinION

To confirm the presence of codon mutation C580Y in *k13*, amplicon sequencing of LAMP reactions by MinION sequencer was performed. LAMP amplicons derived from plasmid DNA harboring partial sequences of *k13* with or without C580Y mutation and clinical samples were purified with Agencourt AMPure XP beads (Beckman Coulter Inc., Brea, CA). For the multiplex and real-time MinION sequencing, a Rapid Barcoding Sequencing Kit (SQK-RBK001, ONT) was used to sequence up to 12 samples on a single flow cell. LAMP amplicons (200 ng each) were measured by Qubit 3.0 Fluorometer (Thermo Fisher Scientific) and simultaneously processed for the barcoding and library preparation using the Rapid Barcoding Sequencing Kit, according to the manufacturer’s instructions. After the MinION Platform QC run, the DNA library was loaded into MinION Flow Cell (FLO-MIN107 R9.5 Version) and the “NC_48Hr_sequencing_FLO-MIN107_SQK-RBK001_plus_basecaller” protocol was initiated using MinKNOW software (ONT, v1.4.2).

### Analysis of MinION data

**Fig. 2:**
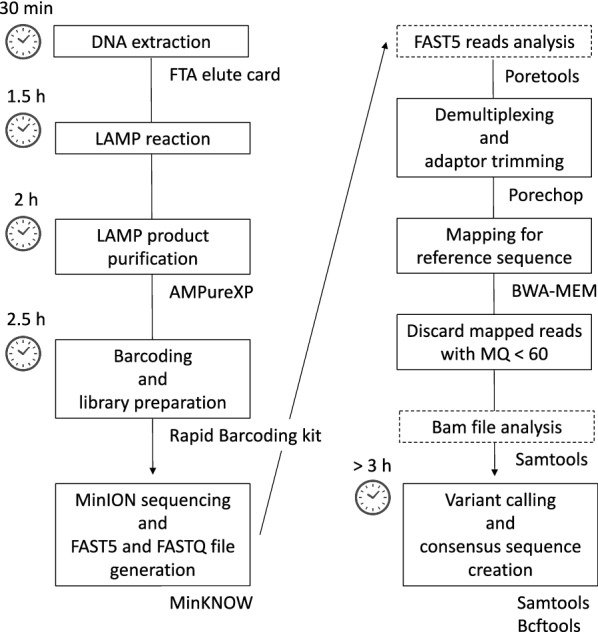
Schematic of the data analysis workflow in this study

Figure 2 shows the workflow of the data analysis in this study. Local basecalling was performed using MinKNOW in real time and automatically. FAST5 reads were collected at 30 min and 48 h after the start of sequencing. FASTQ files were extracted from each collected FAST5 read via Poretools (v 0.6.0). Demultiplexing and adapter trimming was performed using Porechop (v 0.2.2), and each FASTQ per ONT-barcode data was mapped by BWA-MEM (v 0.7.15) using the sequence of *k13* without the C580Y codon mutation between a pair of LAMP primers, F1c (5′-TTG AAT ACC CCT AGA TCA TCA GC-3′) and B1c (5′-AAT GGG AAC AAT TTC CAT ATG CCT-3′). The mapped data with a “MapQuality” < 60 were discarded via Samtools (v 1.5.0). The single nucleotide variant calling from mapped data was obtained via Samtools and BCFtools (v 1.5.0), and cutoff values of 50 for single nucleotide variant quality and read depth were determined. Mapped data were visualized by IGV software (v 2.3.8). FAST5 reads and mapped data were analysed via Poretools and Samtools, respectively.

### *k13* amplification and Sanger sequencing

The Sanger method was used to perform PCR amplicon sequencing to confirm and validate the sequence results of LAMP amplicons via the MinION sequencer for the clinical specimens. For the specific amplification of *k13*, nested PCR according to previously reported protocols were used [7]. Briefly, the first- and second-round PCR amplicons were generated by PrimeSTAR GXL DNA Polymerase (Takara Bio Inc., Kusatsu, Japan) with the following primers: first round forward primers, 5′-CGG AGT GAC CAA ATC TGG GA-3′ and reverse primers, 5′-GGG AAT CTG GTG GTA ACA GC-3′; second round forward primers, 5′-GCC AAG CTG CCA TTC ATT TG-3′ and reverse primers, 5′-GCC TTG TTG AAA GAA GCA GA-3′. Thermal cycling was carried out under the following conditions: 94 °C for 2 min, followed by 40 cycles at 98 °C for 10 s, 55 °C for 30 s, 68 °C for 1 min, with a final extension at 68 °C for 7 min in both the first- and second-round amplifications. The 1000-fold dilution products of the first round of PCR were used as templates for the second round. All second-round PCR products were analyzed using 1.2% (w/v) agarose gel, stained with ethidium bromide, and purified by Agencourt AMPure XP beads (Beckman Coulter). Sanger sequencing of these purified PCR amplicons was performed by Eurofines Genomics Co., Ltd. (Tokyo, Japan).

## Results

### Sensitivity and specificity of LAMP

**Fig. 3:**
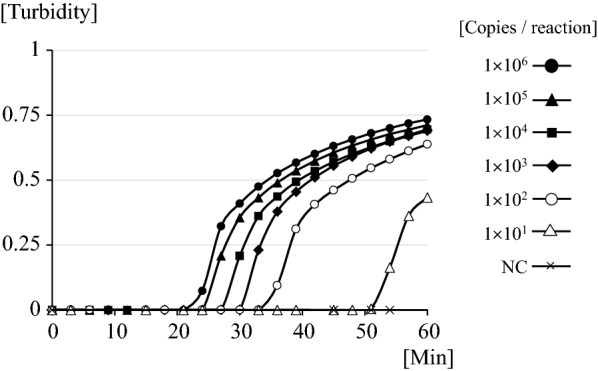
The detection limit of LAMP for *kelch13* of *Plasmodium falciparum* with tenfold serial dilutions of plasmid DNA

A tenfold serial dilution of each plasmid DNA was amplified to determine the lower detection limit of the constructed LAMP assay and the optimal temperature was observed at 62 °C. Figure 3 shows the results of detection based on real-time turbidity; amplification of the target DNA is indicated by the rising curve. The minimum amounts of plasmid DNA on real-time turbidities with LA-200 were 1.0 × 10^1^/reaction within 60 min (Fig. 2). These results were similar to those obtained by observing visual changes within 60 min. Additionally, 1.0 ng of genomic DNA derived from seven other protozoa (*Toxoplasma gondii, Cryptosporidium parvum, Giardia intestinalis, Entamoeba histolytica, Leishmania donovani, Trypanosoma brucei rhodesiense*, and *Trypanosoma cruzi*) were not amplified by the LAMP procedure.

### Clinical evaluation of LAMP assays

Parasitemia was confirmed in 40 blood samples by nested-PCR (33 *P. falciparum*, 6 *P. vivax*, and 1 co-infection with *P. falciparum* and *P. vivax*). All 33 *P. falciparum* and the 1 co-infection cases were positive (n = 34; 100%). None of the 6 *P. vivax* blood samples were amplified with the LAMP primers (Table 2).

**Table 2:** Summary of the results of nested PCR and LAMP amplifications, and the identification of *kelch13* mutations of *P. falciparum*

		LAMP reaction		Identification of mutation			
				PCR and Sanger		LAMP and MinION	
		Positive	Negative	C580Y	Wild type	C580Y	Wild type
Nested PCR							
*P. f*	33	33	0	0	33	0	33
*P. f/P. v*	1	1	0	0	1	0	1
*P. v*	6	0	6	–	–	–	–

*P. f*, *P. falciparum*; *P. v*, *P. vivax*; *P.f/P.v*, *P. falciparum* and *P. vivax* co-infection

### Sequencing of LAMP products by MinION

**Fig. 4:**
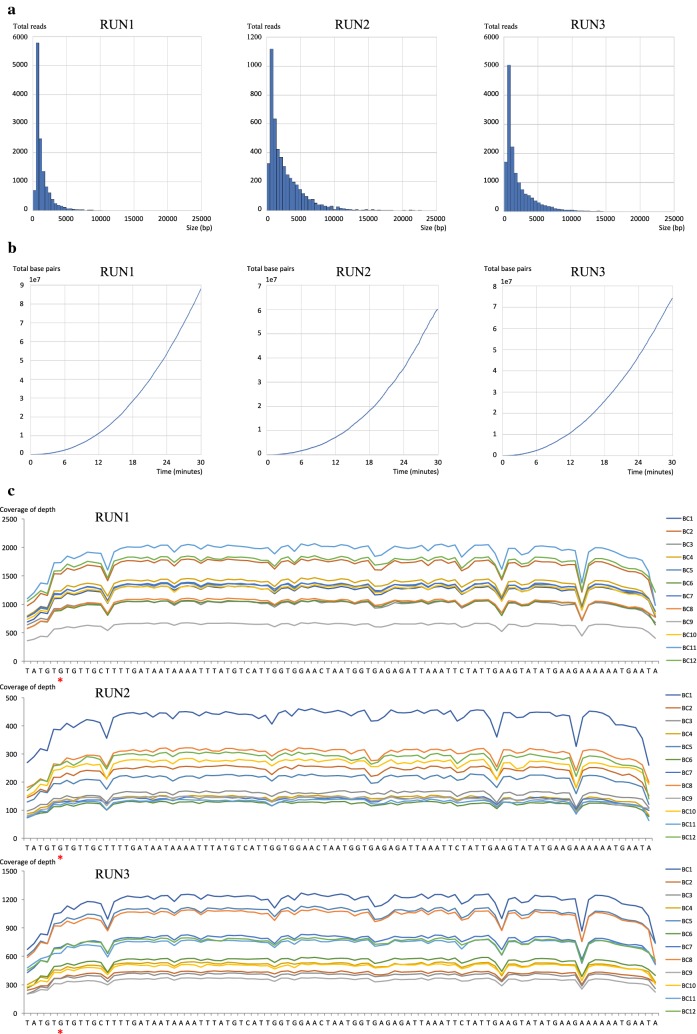
The results of FAST5 reads analysis collected 30 min from the start of MinION sequencing. **a** Histogram of FAST5 read sizes from each MinION sequencing run. **b** Collector’s curve reflecting the total base pairs of sequencing yield over time for each MinION sequencing run. **c** Depth of coverage for each ONT-barcode number and MinION sequencing run

Figure 4 and Additional file 2: Table S1 show the results of FAST5 reads analysis generated 30 min after the start of MinION sequencing. The mean collected read numbers and read bases per run were 10,052 and 2215, respectively. Of the total collected reads, 1.57–7.75% were classified into ONT-barcodes after barcode demultiplexing; however, reads from 35.8 to 70.6% were unclassified. Based on the analysis of the mapped data, a depth of coverage above 50× was achieved at each base on the reference sequence. Because LAMP amplicons contain random repeating structures, a read was mapped as a reference sequence more than once by BWA-MEM.

Table 3 showed the results of mapped data obtained from the analysis workflow using LAMP amplicons with plasmids harbouring the partial sequences of *k13* with or without the C580Y mutation. Variant callings from collected reads were consistent with the plasmid sequences. The average accuracy of the mapped reads of LAMP amplicons generated from plasmids with the codon mutation C580Y was 89.0%, and at the base of the mutation C580Y (position 6/from “G” to “A”), 86.4% reads were correctly matched with “A”, but 13.6% reads were mismatched (Additional file 3: Fig. S2, Additional file 2: Table S2).

**Table 3:** Summary of the MinION sequencing of LAMP amplicons from plasmids

Reference plasmid	Total reads	Mapped reads > MQ60	Coverage	% bases depth > 50	Accuracy (%)	Sequencing result
C580Y	389	793	748.10	100	89.54	C580Y
Wild type	476	822	761.83	100	89.9	Wild type

None of the 34 LAMP amplicons from clinical specimens showed the C580Y mutation based on variant calling from collected reads at 30 min. These results were consistent with the results of PCR amplicon sequencing by conventional Sanger methods (Table 2 and Additional file 2: Table S1). In addition, the same analysis of reads collected 48 h after the start of MinION sequencing were also performed (Additional file 4: Fig. S3 and Additional file 2: Table S3). The results of variant calling were consistent with the results at 30 min.

## Discussion

Although several sophisticated LAMP methods have been developed for the detection of *Plasmodium* parasites from clinical specimens [11, 12, 20–33], none have been designed to detect single nucleotide polymorphisms (SNPs) associated with ART resistance in *P. falciparum*. The LAMP method combined with MinION sequencing method was capable of simultaneously detecting both *P. falciparum* parasitaemia and the codon mutation in *k13* in up to 12 samples at a high level of sensitivity without expensive or large experimental devices. The practical advantage of this method is that it enables real-time monitoring of ART resistance in patients diagnosed with *P. falciparum* malaria, even in areas with limited resources, because the samples can be prepared and sequenced without storage or transfer to a fully-equipped laboratory. Another advantage of this protocol is that sequence data can be stored in the cloud and shared at anytime, anywhere in the world.

The gold standard diagnostic method for malaria to date is the microscopic examination of Giemsa-stained thin or thick blood smears. Several rapid diagnostic tests (RDT), such as the rapid immunochromatographic test, are already manufactured and used throughout the world. However, misdiagnosis is common in microscopic examinations or rapid immunochromatographic tests of mixed infections when a patient has low-density parasitemia or is asymptomatic [34–36]. Consequently, low-density parasitaemia or asymptomatic carriers of *P. falciparum* could be reservoirs for malaria infections [37, 38]. Therefore, highly sensitive diagnostic tools that can detect even low-density parasitaemia are essential for the elimination and accurate epidemiological surveillance of ART-resistant *P. falciparum*. Molecular diagnostic tests such as PCR or LAMP methods are highly sensitive and can detect plasmodium infections even at very low levels of parasitaemia [20, 39–42]. In particular, the detection limit of standard nested-PCR [18], real-time PCR [43], and commercial LAMP assay for malaria (Loopamp™ MALARIA Pan/Pf Detection Kit, Eiken Chemical, Japan) are respectively 6.0, 0.7, and 25.0 parasites/mL, and exceed the detection limit of both microscopy (50–500 parasites/mL) [44, 45] and RDT for *P. falciparum*-specific histidine-rich protein II, *Plasmodium* lactate dehydrogenase, or aldolase (100 parasites/mL) [44]. In this study, the detection limit of the LAMP method for *k13* gene was 1.0 × 10 copies/reaction (corresponding to 12.5 parasite/mL), and the levels of sensitivity and specificity were 100% in the clinical samples. The sensitivity of the LAMP assay is equivalent to previously described LAMP assays (sensitivity ~ 100% and specificity ~ 100%) [11, 12, 20–33].

Using the high specificity of the LAMP method, techniques for genotyping gene polymorphisms without sequence analysis have been developed, including LAMP with FIP and/or BIP primers, which are designed to contain a single-nucleotide polymorphism at each 5′ end (SNPs-LAMP) (http://loopamp.eiken.co.jp/) or insert artificial mutations using FIP and/or BIP primers (ARMS-LAMP) [46, 47]. However, the design of LAMP primers for the above-mentioned protocol is not only quite difficult and limited, but also less sensitive than conventional LAMP assays [48]. In contrast, the LAMP method combined with MinION sequencing can achieve unlimited LAMP primer designs for genotyping polymorphisms.

A diagnostic method for dengue virus via the real-time LAMP method combined with MinION sequencer has been previously developed [16]. However, the multiplex sequence methods for that protocol use barcoded-LAMP FIP primers, which insert a unique 24-base ONT-barcode tag within the F1c sites. In this study, the detection limit of “Barcode-LAMP FIP primers” was one-hundredth that of the “Non-barcoded LAMP FIP primers” (1.0 × 10^3^ copies/reaction vs 1.0 × 10 copies/reaction), and the “Barcode-LAMP FIP primers” can detect only 85.7% of *P. falciparum* parasitaemia cases (Additional file 5: Fig. S4, Additional file 2: Tables S4, S5). In the study by Yamagishi et al., the sensitivity of real-time LAMP in the clinical cases was only 80%, and the LAMP reaction took 90 min. The sensitivity of the above-mentioned LAMP assay was inferior to previously reported LAMP assays for dengue virus (sensitivity ~ 100%) [49–51]. In addition, the protocol took over 2 h to prepare the library using the Ligation Sequencing Kit (SQK-LSK002). In this study, all procedures from DNA extraction to variant calling were completed within 3 h.

It is well documented that MinION reads have a much higher error rate, especially via 1D-chemistry, than conventional Sanger sequencing or other next generation sequencing platforms, including 454, Illumina, and Iontorrent technology [52–55]. The rate of accuracy of 1D-read chemistry with R9.4 flow cell is about 90% (https://nanoporetech.com/). To generate accurate sequences, genomic regions must be read multiple times, with errors eliminated through consensus averaging. Because of the high error rate of MinION, a 50-fold read coverage of the genome positions was sufficient to accurately determine the genotypes. LAMP amplicons are constructed of complicated repeating structures; therefore, a single read generated from MinION contains several multiple base sequences of the target region. This LAMP method combined with MinION sequencing could collect sufficient sequence data within 30 min of starting MinION sequencing. The results of variant calling by this method were completely consistent with the results of Sanger sequencing. Therefore, this procedure is a highly accurate method of variant calling. However, it did not achieve sufficient accuracy for deep sequencing to detect low-frequency mutations within a sample because the average rate of accuracy was 89.4%.

Therefore, if the proportion of the C580Y mutant strain is smaller than that of the wild type strain in the same clinical specimen, the C580Y mutation may not be accurately detected. It is thought that this disparity occurred for the following reasons: the high error rate of MinION reads, the fidelity of the LAMP enzyme, and the mapping algorithm of BWA-MEM. ONT has introduced 1D2-read chemistry with higher precision than 1D-read chemistry (https://nanoporetech.com/), which will improve the precision of the combined LAMP and MinION sequencer method in the future.

This study was limited due to the small number of malaria samples; hence, the performance of the sensitivity and specificity in clinical samples have not been demonstrated. The specificity in clinical samples that contained only *P. vivax* were evaluated. In addition, there were no C580Y mutations in *k13* found among 34 *P. falciparum* isolates collected in 2010 in Indonesia. An accurate evaluation of the sensitivity and specificity of this method requires further investigation in a clinical setting.

The LAMP method combined with MinION sequencing detected SNPs only in the 95 bp region incorporating the C580Y codon mutation in *k13* amplified by the LAMP primers. The other codon mutations in *k13* are reportedly associated with ART resistance. This procedure will be used to detect other mutations for the surveillance of ART resistance in the next study. Due to the flexibility afforded in the design of LAMP primers, this procedure can easily be used to detect other SNPs associated with drug-resistance genes not only in *P. falciparum,* but also in bacteria or fungi.

## Conclusions

In this study, an innovative diagnostic technology to detect the codon mutation C580Y in *k13* of *P. falciparum* was demonstrated. The method, which uses the LAMP assay combined with MinION sequencer, is rapid, simple, and highly sensitive. This procedure could contribute to the epidemiological surveillance of ART-resistant *P. falciparum* and be applied to the analyses of sequence polymorphisms or genotyping. The next step for refining this procedure will be to conduct a clinical evaluation to verify that the sensitivity and specificity are sufficient and consistent in resource-limited endemic regions.

## Additional files

**Additional file 1: Fig. S1.** Sequence alignment of the kelch-propeller domain of human *Plasmodium* parasites and the primer locations for LAMP. *Pf*, *P. falciparum*; *Pv*, *P. vivax*; *Poc*, *P. ovale curtisi*; *Pow*, *P. ovale wallikeri*; *Pm*, *P. malariae*; *Pk*, *P. knowlesi.*

**Additional file 2: Table S1.**  The analysis of FAST5 reads collected 30 min after the start of MinION sequencing. **Table S2.** The results of the visualization analysis and the valiant calling using the reads collected 30 min and 48 h after the start of MinION sequencing. **Table S3.** The analysis of FAST5 reads collected 48 h after the start of MinION sequencing. **Table S4.** Nucleotide sequences of the Barcoded-LAMP primers constructed for *kelch 13* of *Plasmodium falciparum*. **Table S5.** Summary of the results of nested PCR and Barcoded-LAMP amplifications in clinical samples.

**Additional file 3: Fig. S2.** A comparison of visualizations of mapped MinION reads with Sanger sequencing trace data. a: The upper image shows mapped reads from LAMP amplicons generated from plasmid DNA with C580Y as the reference sequence (*kelch13* of wild type; KT956001.1) visualized by igvtools. The lower image is a Sanger sequencing trace of the C580Y allele using the same sample. b: The upper image shows mapped reads from plasmid DNA with the wild type as the reference sequence. The lower image is the Sanger sequencing trace of the wild type allele. Asterisks show the specific sequences located at the codon position of C580Y in *kelch13.*

**Additional file 4: Fig. S3.** The results of FAST5 reads analysis collected 48 h from the start of MinION sequencing. a: Histogram of FAST5 read sizes from each MinION sequencing run. b: Collector’s curve reflecting the total base pairs of the sequencing yield over time for each MinION sequencing run. c: Depth of coverage for each ONT-barcode number and MinION sequencing run.

**Additional file 5: Fig. S4.** The detection limit of Barcode-LAMP assay for *kelch13* of *Plasmodium falciparum* with tenfold serial dilutions of plasmid DNA.
