# Supplementary material for: An innovative diagnostic technology for the codon mutation C580Y in kelch13 of Plasmodium falciparum with MinION nanopore sequencer
Source: Malar J. 2018 May 29;17:217. doi: 10.1186/s12936-018-2362-x (PMC5975513; doi:10.1186/s12936-018-2362-x)
Supplement: Supplementary file 1 — Additional file 1: Fig. S1. Sequence alignment of the kelch-propeller domain of human Plasmodium parasites and the primer locations for LAMP. Pf, P. falciparum; Pv, P. vivax; Poc, P. ovale curtisi; Pow, P. ovale wallikeri; Pm, P. malariae; Pk, P. knowlesi. [file 12936_2018_2362_MOESM1_ESM.pdf]

Fig. S1

```
                    F3                  F2                                    LF
KT956001(Pf)        ATTGGGGGAT ATGATGGCTC TTCTATTATA CCGAATGTAG AAGCATATGA TCATCGTATG
KT961685(Pv)        ATTGGTGGGT ATGATGGGTC GTCTATCATC CCCAATGTGG AAGCCTATGA TCATAGGATG
KT792971(Poc)       ATTGGTGGTT ATGATGGATC ATCAATTATA CCAAATGTTG AGGCGTATGA TCATAGAATG
KT792970(Pow)       ATTGGTGGTT ATGATGGATC ATCAATTATA CCAAATGTTG AGGCGTATGA TCATAGAATG
KT792967(Pm)        ATTGGTGGTT ATGATGGTTC CTCCATTATA CCAAATGTTG AAGCTTATGA TCATCGGATG
XM_002259882(Pk)    ATTGGTGGAT ATGATGGATC GTGTATAATC CCCAATGTGG AAGCATATGA TCATAGAATG

                                                F1
KT956001(Pf)        AAAGCATGGG TAGAGGTGGC ACCTTTGAAT ACCCCTAGAT CATCAGCTAT GTGTGTTGCT
KT961685(Pv)        AAGGCTTGGG TAGAAATCGC CCCCTTGAAT ACGCCTCGAT CTTCCTCCAT GTGTGTAGCC
KT792971(Poc)       AAGGCATGGG TAGAAGTTGC TCCGCTTAAT ACCCCAAGAT CTTCTTCCAT GTGTGTAGCA
KT792971(Pow)       AAGGCATGGG TAGAAGTTGC TCCTCTTAAT ACCCCAAGAT CTTCTTCTAT GTGTGTCGCA
KT792967(Pm)        AAAGCATGGG TAGAAATTGC ACCTTTAAAT ACTCCAAGAT CTTCATCCAT GTGTGTAGCT
XM_002259882(Pk)    AAGGCTTGGG TAGAAATTGC CCCATTGAAT ACTCCAAGAT CTTCGTCCAT GTGTGTAGCC

KT956001(Pf)        TTTGATAATA AAATTTATGT CATTGGTGGA ACTAATGGTG AGAGATTAAA TTCTATTGAA
KT961685(Pv)        TTTGACAACA AAATATATGT CATCGGTGGG ACCAATGGAG AAAGACTAAA TTCGATCGAA
KT792971(Poc)       TTTGATAATA AAATATATGT TATTGGTGGA ACAAATGGAG AGAGATTAAA TTCAATAGAA
KT792971(Pow)       TTTGATAATA AAATTTATGT TATTGGTGGA ACAAATGGAG AGAGATTAAA TTCAATAGAA
KT792967(Pm)        TTTGACAATA AAATATATGT TATAGGTGGA ACAAATGGAG AGAGATTAAA TTCAATTGAA
XM_002259882(Pk)    TTTGAAAACA AAATTTATGT CATCGGTGGA ACGAATGGAG AAAGATTAAA TTCGATTGAA

                                                                  B1
KT956001(Pf)        GTATATGAAG AAAAAATGAA TAAATGGGAA CAATTTCCAT ATGCCTTATT AGAAGCTAGA
KT961685(Pv)        GTGTATGATG AAAAGATGAA CAAGTGGGAG CAATTTCCGT ACGCCTTGTT AGAAGCCAGA
KT792971(Poc)       GTATATGATG AAAAAATGAA TAAATGGGAA CAATTCCCTT ATGCACTTTT AGAAGCTAGA
KT792971(Pow)       GTATATGATG AAAAAATGAA TAAATGGGAA CAATTCCCTT ATGCACTTTT AGAAGCTAGA
KT792967(Pm)        GTATATGAAG AAAAAATGAA TAAATGGGAA CAATTTCCAT ATGCATTATT AGAAGCTAGA
XM_002259882(Pk)    GTGTATGATG AAAAGATGAA CAAATGGGAG CAATTTCCGT ACGCTTTGTT AGAAGCCAGA

                                                    B2                           B3
KT956001(Pf)        AGTTCAGGAG CAGCTTTTAA TTACCTTAAT CAAATATATG TTGTTGGAGG TATTGATAAT
KT961685(Pv)        AGCTCAGGGG CAGCTTTTAA CTACCTAAAT CAGATATATG TCGTTGGGGG GATTGACAAC
KT792971(Poc)       AGTTCAGGTG CAGCTTTTAA TTATCTAAAT CAAATATACG TTGTTGGAGG TATTGATAAT
KT792971(Pow)       AGTTCTGGTG CAGCTTTTAA TTATCTAAAT CAAATATACG TTGTGGGAGG TATTGATAAT
KT792967(Pm)        AGCTCAGGTG CAGCTTTTAA TTACTTAAAT CAAATATATG TCGTTGGAGG TATTGATAAT
XM_002259882(Pk)    AGTTCAGGTG CAGCTTTTAA CTACCTAAAT CAGATATATG TTGTTGGGGG TATTGACAAC
```
