# Supplementary figures and images for: An innovative diagnostic technology for the codon mutation C580Y in kelch13 of Plasmodium falciparum with MinION nanopore sequencer
Source: Malar J. 2018 May 29;17:217. doi: 10.1186/s12936-018-2362-x (PMC5975513; doi:10.1186/s12936-018-2362-x)

Fig. S2

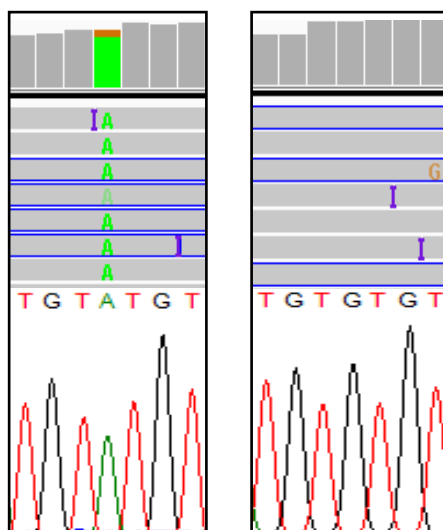

Supplement: Supplementary file 3 — Additional file 3: Fig. S2. A comparison of visualizations of mapped MinION reads with Sanger sequencing trace data. a: The upper image shows mapped reads from LAMP amplicons generated from plasmid DNA with C580Y as the reference sequence (kelch13 of wild type; KT956001.1) visualized by igvtools. The lower image is a Sanger sequencing trace of the C580Y allele using the same sample. b: The upper image shows mapped reads from plasmid DNA with the wild type as the reference sequence. The lower image is the Sanger sequencing trace of the wild type allele. Asterisks show the specific sequences located at the codon position of C580Y in kelch13. [file 12936_2018_2362_MOESM3_ESM.pdf]

Fig. S3  
a

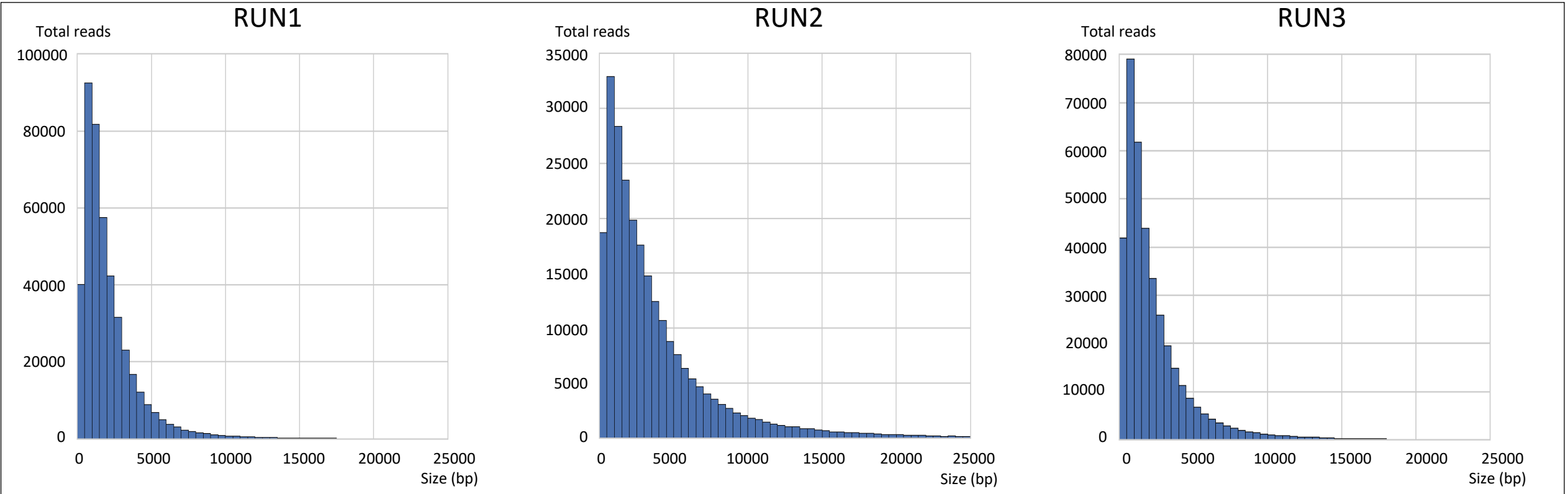

b

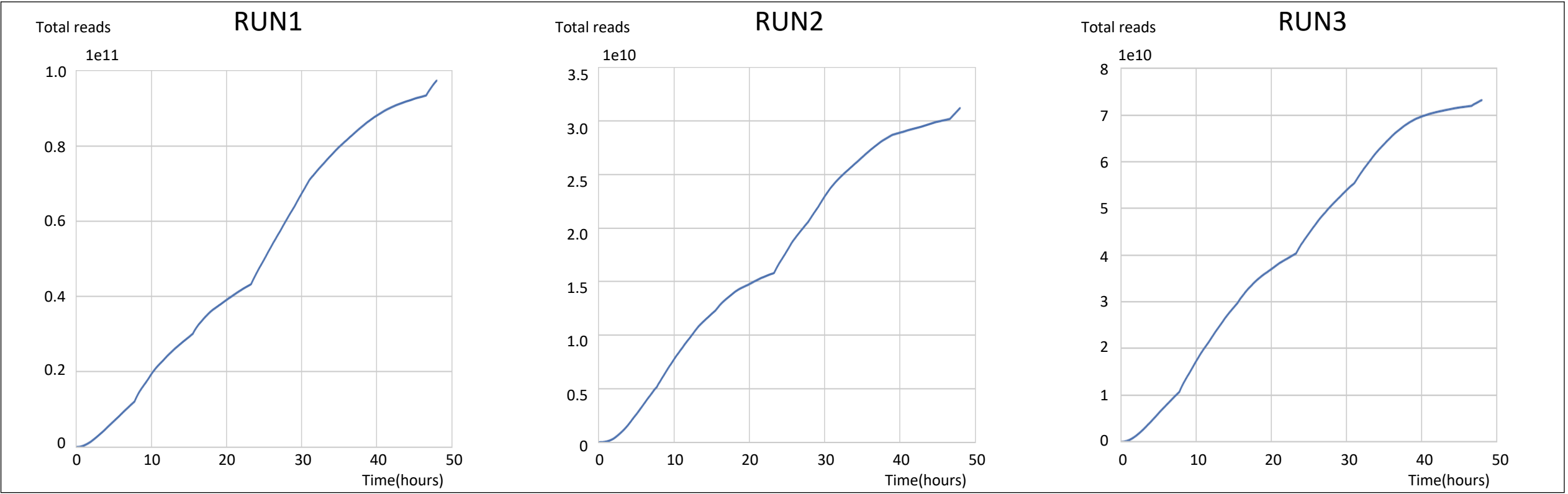

c

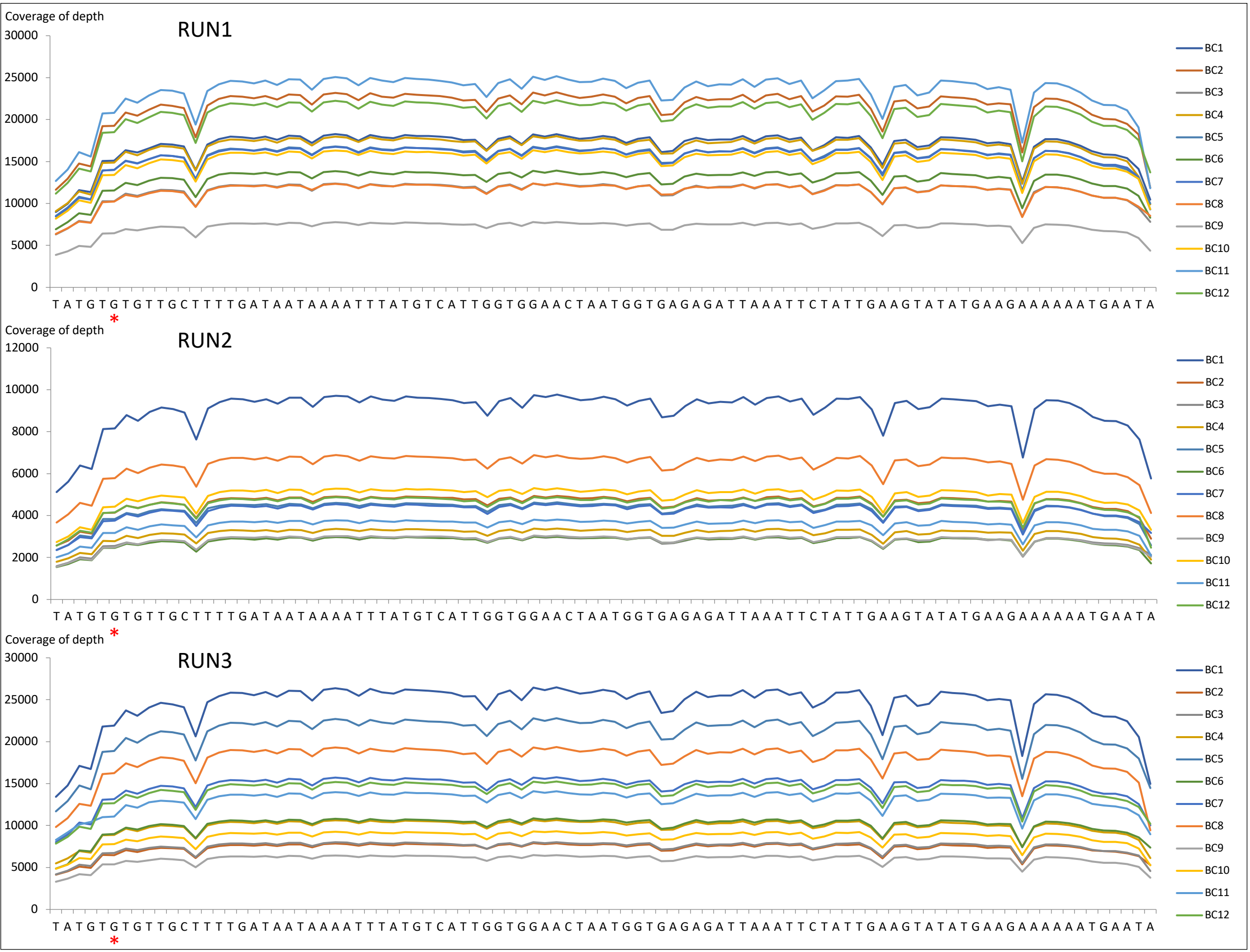

Supplement: Supplementary file 4 — Additional file 4: Fig. S3. The results of FAST5 reads analysis collected 48 h from the start of MinION sequencing. a: Histogram of FAST5 read sizes from each MinION sequencing run. b: Collector’s curve reflecting the total base pairs of the sequencing yield over time for each MinION sequencing run. c: Depth of coverage for each ONT-barcode number and MinION sequencing run. [file 12936_2018_2362_MOESM4_ESM.pdf]

Fig. S4

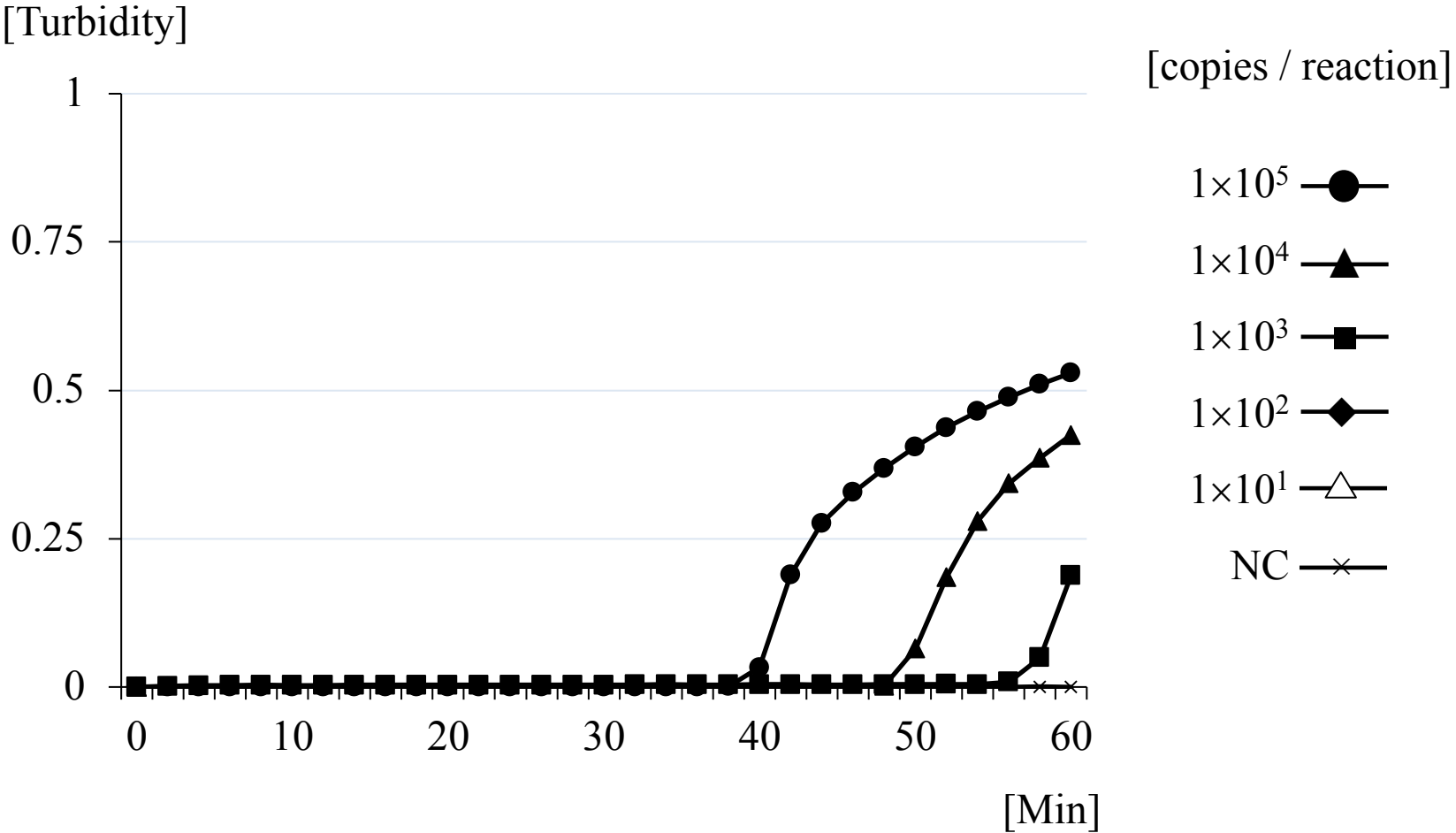

Supplement: Supplementary file 5 — Additional file 5: Fig. S4. The detection limit of Barcode-LAMP assay for kelch13 of Plasmodium falciparum with tenfold serial dilutions of plasmid DNA. [file 12936_2018_2362_MOESM5_ESM.pdf]
